# *ScolioClass*: data-driven development of a new classification tool to evaluate adolescent idiopathic scoliosis optically diagnosed

**DOI:** 10.3389/fdgth.2025.1633612

**Published:** 2025-12-18

**Authors:** Saša Ćuković, Mihai Neghina, Radu Emanuil Petruse, Vanja Luković, Dijana Stojić, Yiying Zou

**Affiliations:** 1 ETH Zurich, Institute for Biomechanics, Laboratory for Movement Biomechanics, Zurich, Switzerland; 2 Lucian Blaga University of Sibiu, Department for Computer Science and Electrical Engineering, Sibiu, Romania; 3 Lucian Blaga University of Sibiu, Department for Industrial Engineering and Management, Sibiu, Romania; 4 University of Kragujevac, Faculty of Technical Sciences, FTN Čačak, Čačak, Serbia; 5 The Hong Kong Polytechnic University, Department of Biomedical Engineering, Kowloon City, Hong Kong SAR, China

**Keywords:** adolescent idiopathic scoliosis (AIS), non-invasive diagnosis, *ScolioSIM*, *ScolioClass* - classification tool, optical measurement, MATLAB

## Abstract

Adolescent idiopathic scoliosis (AIS) is traditionally assessed and classified using radiographic methods that rely on Cobb angle measurements and qualitative curve modifiers, exposing patients to repeated radiation and offering limited sensitivity to subtle three-dimensional (3D) deformities. We developed *ScolioClass*, a non-invasive, data-driven classification tool that harnesses 3D optical surface scanning and continuous indices, capturing curvature severity, directionality, and sagittal balance, to evaluate spinal deformities in 94 patients with AIS. By comparing *ScolioClass* descriptions with the established Lenke classification, we observed a statistically significant association (*χ*^2^ ≈ 29.0, df = 6, *p* < 0.001) with 72.3% overall agreement. A significant association was also found between sagittal modifiers and *ScolioClass* kyphosis–lordosis categories (*χ*^2^ ≈ 48.4, df = 3, *p* < 0.0001) with 68.1% agreement. Notably, *ScolioClass* detected mild curves and lordotic patterns that were often overlooked by Lenke criteria. These findings demonstrate that *ScolioClass* provides radiation-free, quantitative 3D assessment of AIS with potential for automated analysis and individualized treatment planning. Further validation is warranted for clinical integration.

## 1 Introduction

**Figure 1:**
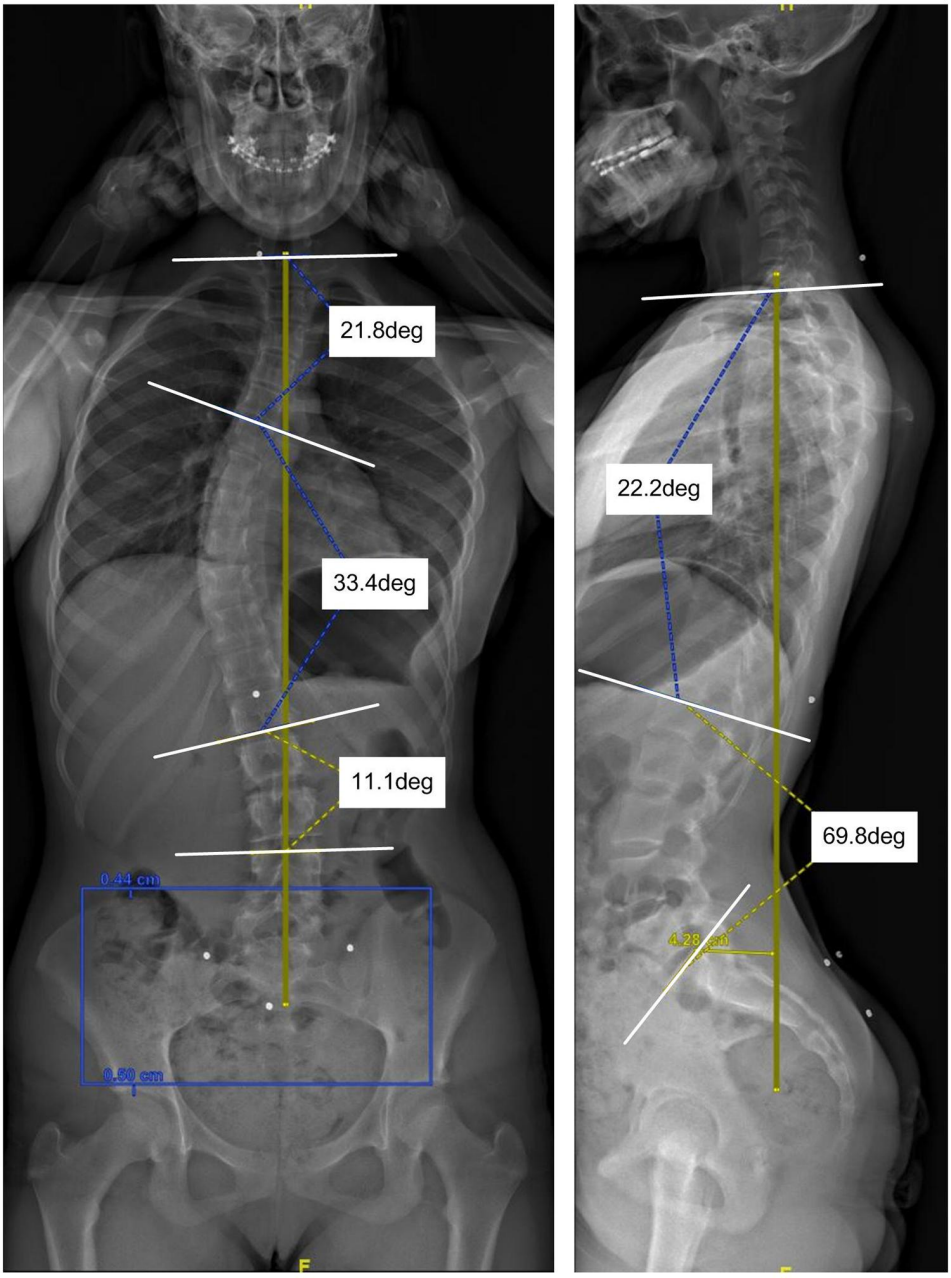
X-ray image of an AIS subject with basic radiographic measurements – 3 cobb angle measurements.

Scoliosis is a complex three-dimensional (3D) spinal deformity that influences patients' health, physical appearance, and quality of life (1). It affects 0.5%–5.2% of the global population, with 2%–4% classified as idiopathic scoliosis of unknown etiology (2) and nearly 80% of these cases are adolescent idiopathic scoliosis (AIS) (3). AIS is typically diagnosed using coronal radiographic imaging, with the Cobb method serving as the clinical gold standard (4). The Cobb angle is defined as the angle between lines drawn along the upper and lower endplates of the most tilted vertebrae above and below the curve apex, respectively (5). Clinically, scoliosis is defined as a lateral spinal curvature with a Cobb angle of 10° or greater, measured on upright standing radiographs (5) (Figure 1). Several classification systems based on the Cobb angle have been developed to guide the diagnosis and treatment. The King system focuses on frontal plane curvature (6), while the more comprehensive Lenke system incorporates both coronal and sagittal planes for surgical planning (7). The Peking Union Medical College (PUMC) system is simpler but less commonly applied (8, 9).

A major limitation of traditional radiographic assessment methods is the risk of radiation exposure, particularly for young populations (10–12). Since patients with AIS are typically examined every 3–6 months to monitor curve progression or treatment effectiveness, repeated exposure to ionizing radiation can significantly increase the long-term risk of malignancy (13). Moreover, conventional classification systems are primarily based on two-dimensional (2D) imaging, which may fail to accurately capture the 3D nature of the deformity (6, 7). In addition, their implementation in clinical practice requires highly experienced clinicians and is associated with considerable inter- and intra-observer variability (6, 14).

To reduce radiation exposure, non-ionizing imaging methods such as 3D raster stereography have been developed (15, 16). These techniques estimate spinal deformity by analyzing the back surface using light, with the goal of inferring internal spinal alignment (ISA) from external back profiles (17). However, their accuracy in establishing a reliable correspondence with ISA remains limited and requires further validation against radiographic standards (18).

To address these limitations, we propose *ScolioClass*, a novel 3D classification system for AIS based on non-invasive optical scanning and statistical analysis. Built upon the 3D optical digitalization platform *ScolioMedIS* (19), *ScolioClass* integrates external surface data with estimated 3D ISA and biplanar x-ray alignment to enable comprehensive, multi-planar analysis. It introduces three continuous indices—STD-index (severity), *v*-index (location), and kl-index (sagittal balance)—for radiation-free, objective classification of spinal deformity. This data-driven approach captures the 3D geometry of the spine, surpassing traditional 2D methods in both accuracy and clinical applicability. By enhancing early detection and supporting personalized treatment planning, *ScolioClass* presents a significant advancement in AIS classification.

Traditional radiographic and 2D classification methods, while widely used, are restricted by radiation risks, observer variability, and the inability to capture the true 3D geometry of scoliosis. In contrast, non-ionizing 3D optical digitalization provides rich patient-specific data, including vertebral centroids and spinal alignments, which allow a more objective analysis. Because these data are high-dimensional and continuous, their interpretation requires statistical processing rather than fixed manual rules. For this reason, our approach is data-driven: it relies on empirical 3D measurements and mathematical/statistical analysis to extract curvature features, evaluate deformity severity, and group patients into meaningful categories. This strategy bridges the gap between safe non-ionizing acquisition and accurate 3D classification, without resorting to repeated x-ray exposure.

In this study, we describe the development of a novel MATLAB tool, *ScolioClass*, designed to classify scoliosis based on 3D spinal deformity alignments estimated from non-ionizing optical scans of patients' 3D back surfaces. Using data-driven techniques, we extract geometric features from the spinal curves and cluster them into clinically meaningful groups that reflect deformity characteristics and severity. This work represents a step towards a more objective and comprehensive 3D classification system for AIS.

The remainder of this paper is organized as follows. Section 2 provides information on the materials and methods, describing the data acquisition and processing, including the fine interpolation and the computation of statistical indices. Section 3 presents the experimental results and validation against well-established methods. Finally, section 4 concludes the paper and outlines possible generalizations and directions for future work.

## 2 Materials and methods

**Figure 2:**
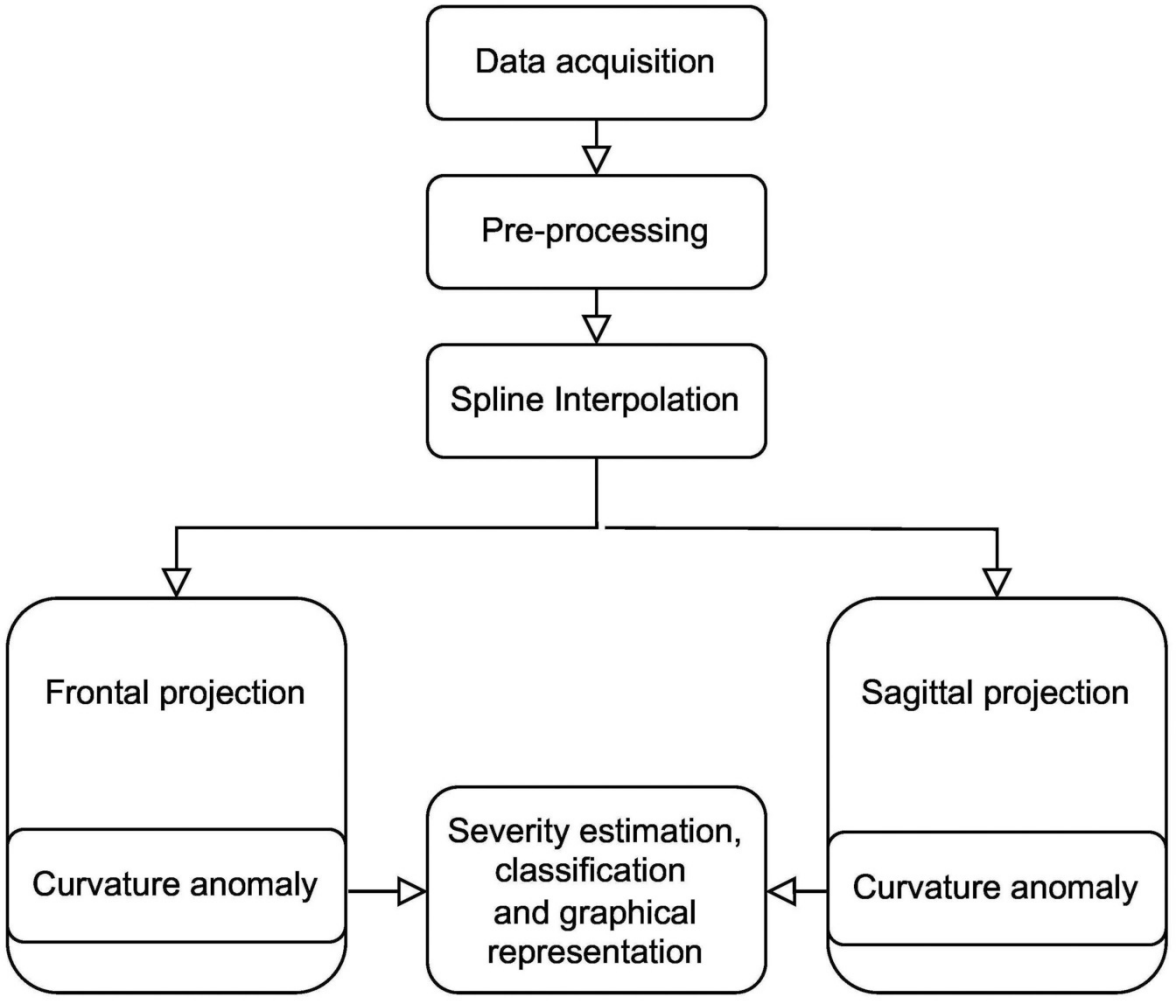
Workflow of the analysis and classification inside the *ScolioClass* app.

After patient-specific data acquisition using optical and radiographic imaging, data analysis begins as depicted in Figure 2. During the pre-processing phase, the 3D coordinates (X, Y, Z, and rotation angles) for each vertebra and intervertebral disc centroids were identified on a digital back surface using the *ScolioSIM* module of the *ScolioMedIS* system (19). Subsequently, a spline with a flexion point distance of 1 mm is interpolated through the coordinates obtained in the previous step using MATLAB 2022. This 3D spline was then projected onto the frontal and sagittal planes to detect curvature anomalies, as explained in (20). The final step involves the estimation of the spinal deformation severity, visual representation of the deformity values, and classification. Each of the above-mentioned steps is described in detail (Sections 2.1 to 2.3).

### 2.1 Participants

In this study, we retrospectively analysed previously collected samples of 94 AIS patients (18). They were recruited according to the following inclusion criteria: age 8 years until achieving skeletal maturity (approx. 16–18 years) and AIS diagnosed (without any known vertebral deformity or neurogenic cause). Exclusion criteria were all non-idiopathic scoliosis cases: i.e., early onset scoliosis (<8 years), syndrome-associated, neurological patients (cerebral palsy, syringomyelia, etc.) or with a structural deformity (e.g., hemi-vertebra), inability to follow the procedures of the study, e.g., due to psychological disorders, dementia of the participant, etc. Belonging to this group, there are 83 female and 11 male participants (mean age is 13.2 ± 2.3 years, weight 49.0 ± 10.3 kg, and height 158.7 ± 11.2 cm).

As the standard of care, a regular clinical examination was conducted using the biplanar EOS x-ray device to evaluate the deformity curve. Additionally, a 3D optical and markerless scanning was performed using an optical scanner to digitalize the patient's back surface at the IRCCS Istituto Ortopedico Galeazzi, Milan, Italy (18). For this study, we selected 94 fully anonymous samples with the best matching between optical surface scans and DICOM images, as they were collected at different time points.

### 2.2 Data acquisition and optical data processing

**Figure 3:**
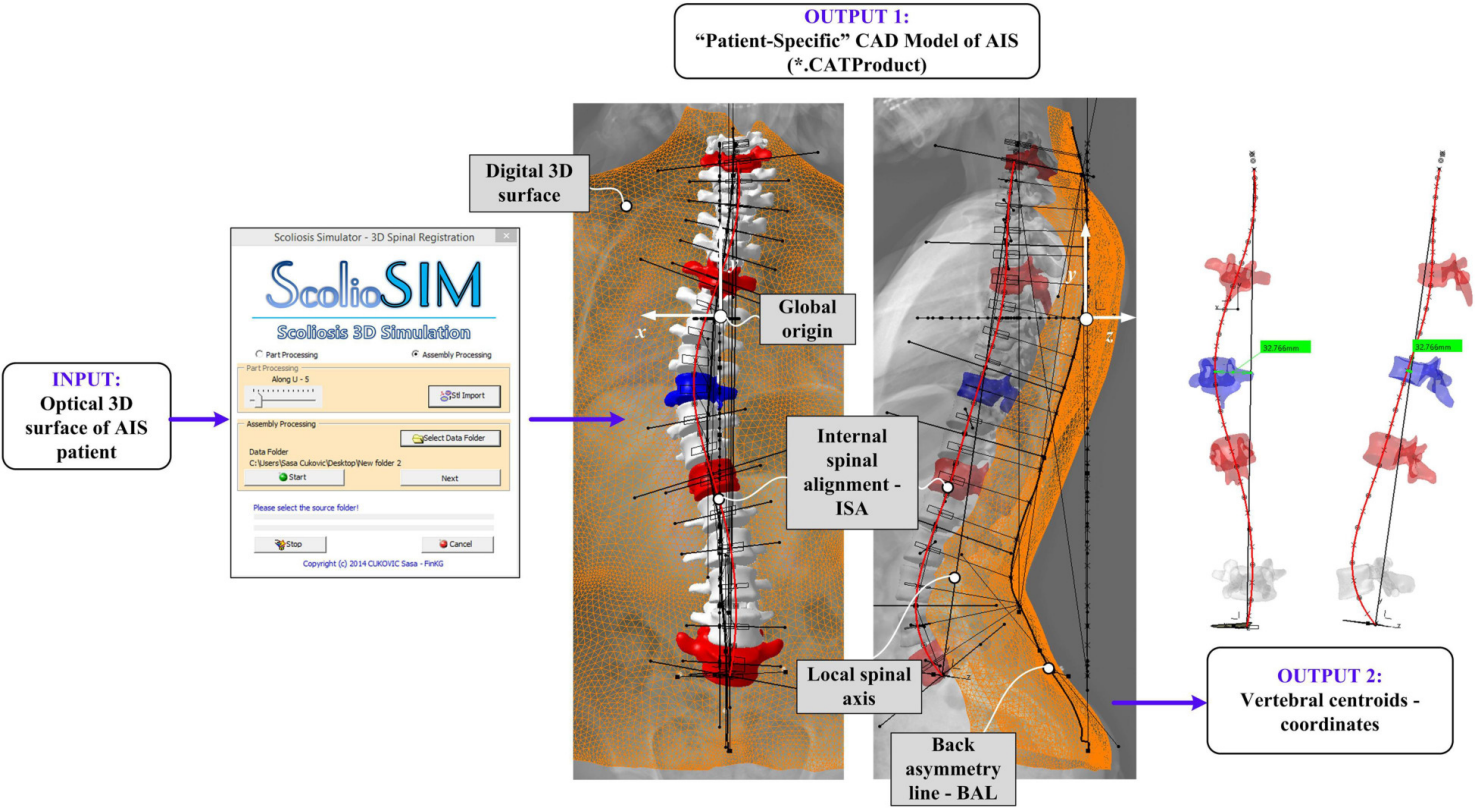
Patient-specific 3D deformity model generated by the ScolioSIM tool for the subject illustrated in figure 1.

To non-invasively acquire patient-specific spinal data for the development of the proposed AIS classification solution (*ScolioClass*), a 3D optical digitalization was employed. In this step, the optical scanner captured the dorsal surface of the patients (18). Anatomical landmarks and back asymmetry line (BAL) (the 3D curve that passes through spinous processes) are manually drawn, while the internal spinal alignment (ISA) (the 3D curve that passes through centroids of intervertebral discs and vertebral bodies) from L5/S1 to C7 curves is drawn based on biplanar x-ray images registered on the back surface. Biplanar x-ray images were manually aligned towards 3D optical surfaces based on visible prominent points, and 2D spline-type curves were used to approximate ISA. This registration process ensures that ISA reflects the true anatomical position of the spine, as seen in the biplanar x-ray, allowing for more precise measurements and assessments of spinal deformities. The 3D coordinates of the key anatomical landmarks of the back (C7, DL, DM, DR, S, ITL, ISL), BAL, ISA, together with the 3D optical scan and the generic spine 3D model (GS 3D model) are input for the *ScolioSIM* tool (21). The *ScolioSIM* (22, 23) is a part of the *ScolioMedIS* (19) system that allows an online, non-invasive 3D quantification of AIS deformities (Figure 3).

The *ScolioSIM* imports the back surface as *.stl file and *.text files of anatomical landmarks, BAL, and ISA into the PLM system CATIA v5 and performs calculations of deformity indicators (intrinsic and extrinsic) and generates several parameters measured in all three body planes, such as transpositions, rotations, inflexion points, Cobb angles, the position of apex vertebra, etc. The *ScolioSIM* tool loads the generic 3D spine model, rescales it according to the back shape size, finds the apex and end vertebrae, and performs rigid registration of vertebrae on ISA to obtain the patient-specific 3D spine model (PS 3D spine model). Figure 3 illustrates the PS 3D spine model of the subject from Figure 1 and its optical surface processed by the *ScolioSIM* tool.

Subsequently, the *ScolioSIM* tool was utilized to identify the position of each vertebra from L5/S1 to C7, forming the ISA, which is the 3D curve that passes through the centroid of each vertebral body. This curve further facilitated the calculation of 3D spinal curvatures and inflexion points. 3D coordinates of each vertebral centroid and transpositions from the local spinal axis were then input to build the classes in MATLAB.

### 2.3 *ScolioClass* app - MATLAB program

The analysis and representation of all the ISA samples generated using *ScolioSIM* have been continued in MATLAB 2022 with two goals (24):

- To intuitively display geometrical information of the ISA curve in appropriate planes to allow medical professionals a good overview of the data, both in 2D and 3D.
- To automatically analyse the patients’ spinal data and to represent the estimated intensity of various curvature abnormalities using a comprehensive colour code.

As a step further, the analysis includes a language-based description of the estimated irregularities of the deformity curve, not intended to replace the official medical diagnosis, but to aid in the identification of the AIS classes.

#### 2.3.1 Pre-processing

The input of the pre-processing step, as shown in Figure 2, is the list of 3D coordinates of vertebral and intervertebral centroids and their angular orientation on the ISA curve. To ensure consistent analysis, the spine data was scaled to a C7-L5 vertebral height of 500 mm. It is also adjusted so that C7 and L5 align vertically and translated so that L5 is at the origin of the axis system (0,0,0).

#### 2.3.2 Interpolation

**Figure 4:**
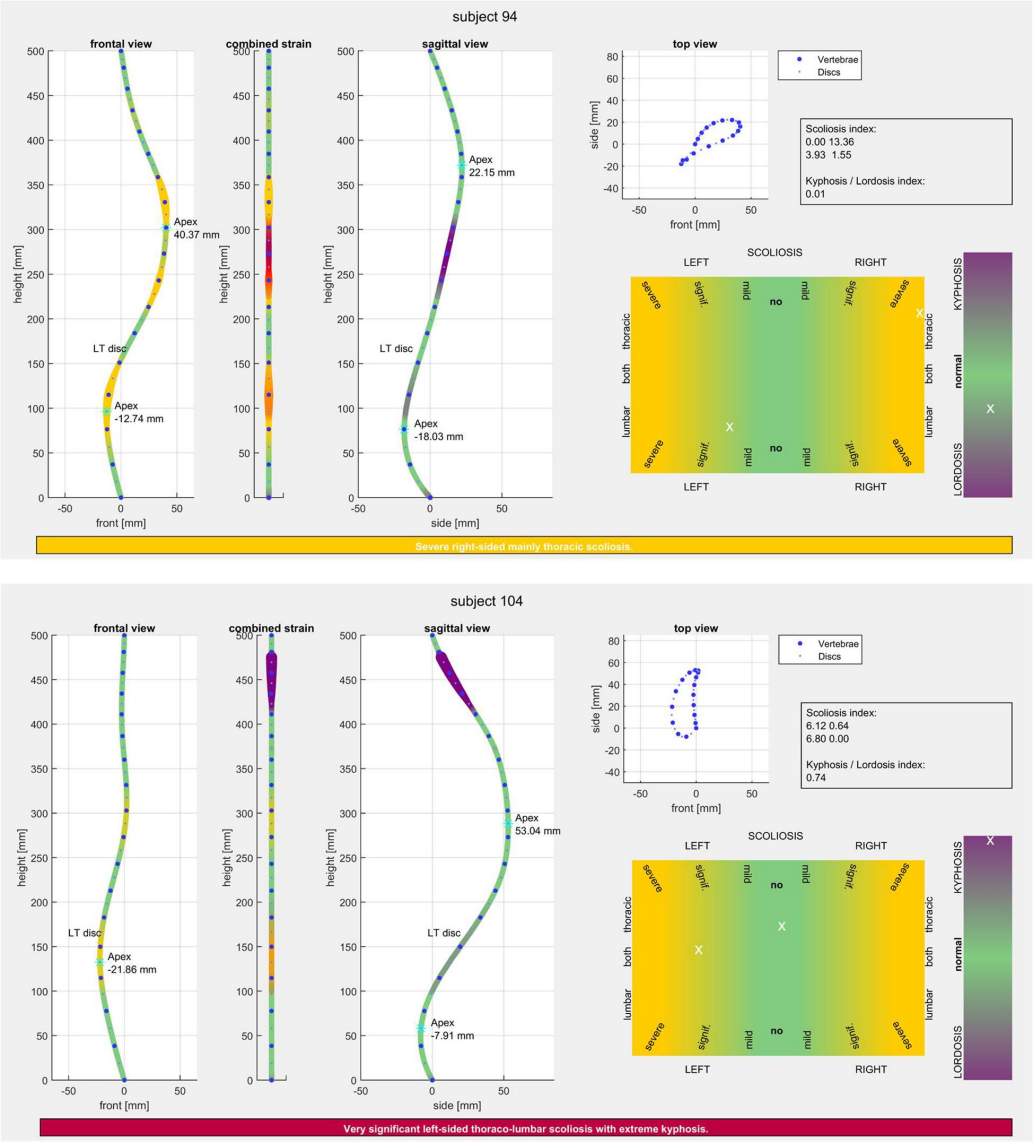
MATLAB 2D representations of the ISA deformity curves for two different subjects (severe scoliosis vs. severe kyphosis) and the classes and sub-classes estimation, including natural language text.

Each ISA curve is computed in MATLAB 2022 using fine 3D cubic spline interpolation with points spaced 1 mm apart. While it is obvious that the spine is composed of vertebrae and vertebral discs, the interpolated curve serves as a smooth connection between them to estimate the strain on the spine (Figure 4). The mathematical curvature of the finely interpolated curve was computed and evaluated by the authors in their previous paper (20).

#### 2.3.3 AIS classes development

The MATLAB 2022 was also employed to compute the frontal projection of the ISA interpolated curve, which is then used for the AIS analysis in two planes.

The projected curves are further divided into the thoracic and lumbar regions, depending on their position relative to the LT disc (disc in which the spine transits from the lumbar to thoracic regions) (Figure 4). Since the projection is expected to have all samples in a vertical line, one statistically relevant measure of displacement is the standard deviation, and it is computed in the thoracic-left (std_TL_), thoracic-right (std_TR_), lumbar-left (std_LL_) and lumbar-right (std_LR_), resulting in 4 values. If the spine deformity is such that no points are in one (or more) of these categories, the standard deviation is considered as 0 (see the Appendix).

$$\mathrm{STD}=\left[\begin{array}{cc} \mathrm{st}d_{\mathrm{TL}} & \mathrm{st}d_{\mathrm{TR}}\\ \mathrm{st}d_{\mathrm{LL}} & \mathrm{st}d_{\mathrm{LR}} \end{array}\right]$$ (1)

For representational purposes, the maximum displacements to the left and right of any vertebra or disc centroids are also stored and will be further referred to as apexes. For the kypho-lordosis analysis, the sagittal projection (seen from the left side of the patient) of the interpolated curve is used. Since the projection is expected to have a big thoracic curve with relatively small and constant negative mathematical curvature, followed by a smaller lumbar curve with relatively larger positive mathematical curvature, here, a good basic measure of the displacement is the mean value of the projection points. For representational purposes, the maximum thoracic and lumbar displacements are also stored.

Table 1 shows the interpretation of the values in the 6 classes estimation. It is worth emphasizing that all 4 of the std indices can conceivably be any positive value, and new boundaries could be easily defined if the medical professionals consider them better suited. Furthermore, the terminology chosen to describe the AIS classes in the class estimation is descriptive and not associated with established medical terms (for e.g., classes according to Cobb angle values).

**Table 1:** AIS index interpretations in the class estimation, interpretation *s* of s index in the class estimation, interpretation of *v* index in the class estimation, interpretation of kl index in the class estimation.

Std value range	Text interpretation	*s* index value range	Text interpretation	*v* index value range	Text interpretation	kl. index value range	Text interpretation
[ 0, 2.5)	No scoliosis	s<−0.5	Left-sided scoliosis	v∈[−1.00,−0.60)	Lumbar scoliosis	kl∈[−1.00,−0.35)	Extreme lordosis
[2.5, 4.0)	Mild scoliosis	|s|≤0.5	Both-sided scoliosis	v∈[−0.60,−0.25)	Mainly lumbar scoliosis	kl∈[−0.35,−0.20)	Significant lordosis
[4.0, 6.5)	Slightly significant scoliosis	s>+0.5	Right-sided scoliosis	v∈[−0.25,+0.25]	Thoraco-lumbar scoliosis	kl∈[−0.20,0)	Slight lordosis
[6.5, 10.0)	Very significant scoliosis	s index value range	Text interpretation	v∈(+0.25,+0.60]	Mainly thoracic scoliosis	kl∈[0,+0.30]	Normal spine
[10.0, 14.5)	Severe			v∈(+0.60,+1.00]	Thoracic scoliosis	kl∈(+0.30,+0.50]	Slight kyphosis
[14.5, ∞)	Extremely severe					kl∈(+0.50,+0.65]	Significant kyphosis
						kl∈(+0.30,+1.00]	Extreme kyphosis

For the class estimation, the largest AIS index value generates the words of the text interpretation, as seen in Table 1. If there is at least mild scoliosis, the sidedness and vertical index are also computed to localize the scoliosis. In deciding whether the scoliosis is left-sided or right-sided, the sidedness index *s* is computed as:

$$s=\frac{\left(\mathrm{st}d_{\mathrm{TR}}+\mathrm{st}d_{\mathrm{LR}})-(\mathrm{st}d_{\mathrm{TL}}+\mathrm{st}d_{\mathrm{LL}}\right)}{\mathrm{st}d_{\mathrm{TR}}+\mathrm{st}d_{\mathrm{LR}}+\mathrm{st}d_{\mathrm{TL}}+\mathrm{st}d_{\mathrm{LL}}}\in [-1,1]$$ (2)

For vertical localisation of the scoliosis, the vertical index *v* is computed as:

$$v=\frac{\left(\mathrm{st}d_{\mathrm{TR}}+\mathrm{st}d_{\mathrm{TL}})-(\mathrm{st}d_{\mathrm{LR}}+\mathrm{st}d_{\mathrm{LL}}\right)}{\mathrm{st}d_{\mathrm{TR}}+\mathrm{st}d_{\mathrm{TL}}+\mathrm{st}d_{\mathrm{LR}}+\mathrm{st}d_{\mathrm{LL}}}\in [-1,1]$$ (3)

The kyphosis/lordosis index kl is computed from the means of the thoracic and lumbar points of the sagittal projection of AIS curve, denoted $m_{T}$ and $m_{L}$ in the Equation (4):

$$\mathrm{kl}=\frac{m_{T}+m_{L}}{m_{T}-m_{L}}\in [-1,1]$$ (4)

When considering that all std values are positive, whereas $m_{L}$ is negative, it is easy to notice that the kl index is computed in a similar way to the *s* and *v* indices However, because the normal spine is not balancing the two segments of the spine, the interpretation is shifted around a positive value.

### 2.4 Graphical data representation

**Figure 5:**
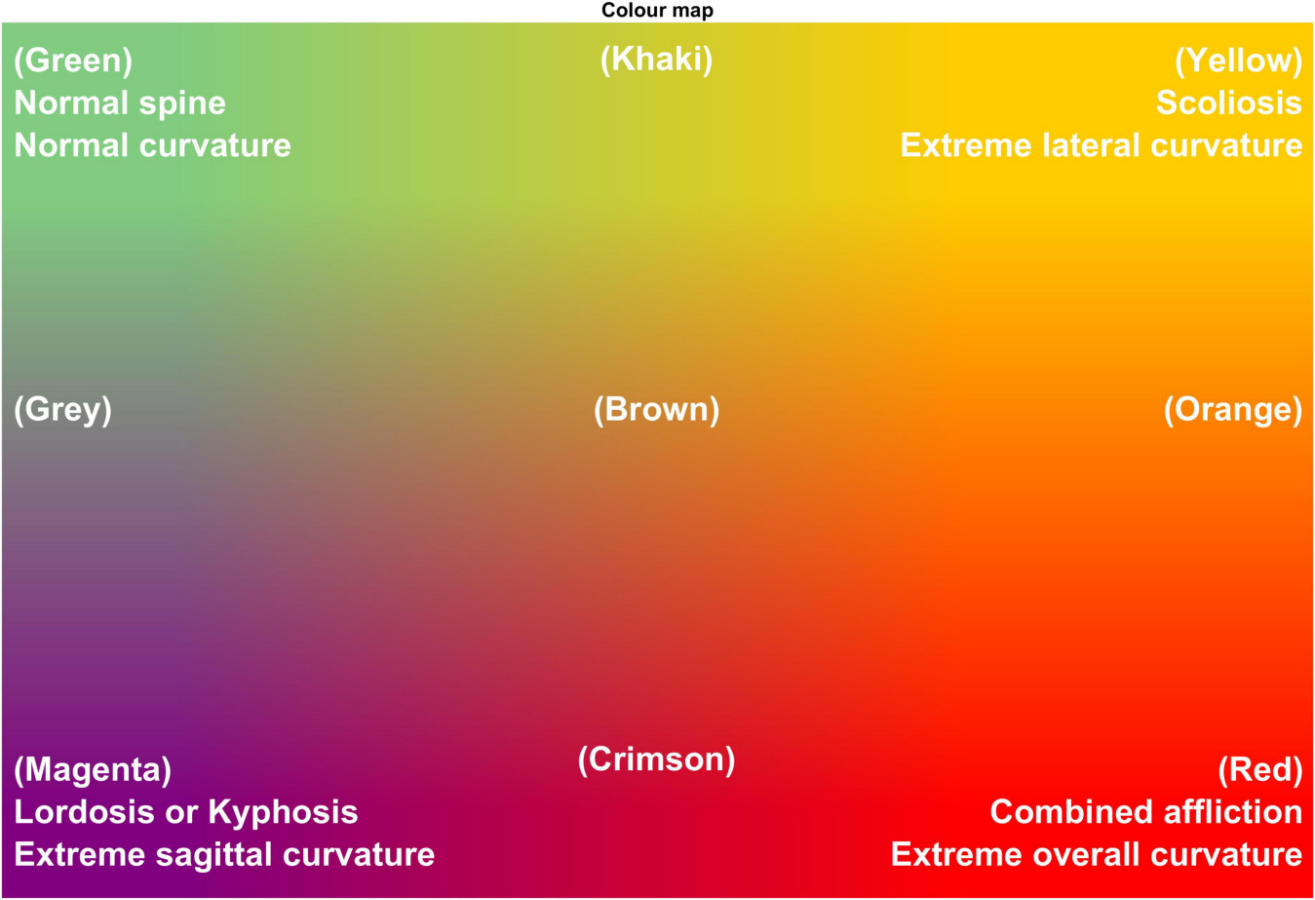
Colour scheme interpretation – visualisation of mathematical curvature strain (25).

Figure 5 illustrates a part of *ScolioClass* interface and contains 2D representations of three ISA projections in frontal, sagittal and axial views. For all projections, the position of the vertebrae and discs centroids are displayed with large blue dots and small blue dots, respectively. The LT disc is also marked, representing the transitioning point between the lumbar and thoracic regions. The apexes in the sagittal projection are denoted *ApexT* in the thoracic region and *ApexL* in the lumbar region. In the frontal projection, there can be one right and one left-side apex, both denoted as *ApexS*. If the spine does not have displacements in the frontal projection, the corresponding side apex does not exist. The graphical representations of the values and interpretation of classes defined in 2.2.3 are presented in two heat maps, with green to yellow for scoliosis and green to magenta for kyphosis/lordosis. It is worth emphasising that the scoliosis representations contain two × marks, for the left and right side, whereas the kyphosis/lordosis representation only contains one × mark.

The scoliosis index box contains values of AIS standard deviation indices, and below is the value of the kyphosis/lordosis index. The AIS values in the upper row are thoracic-left $\left(\mathrm{st}d_{\mathrm{TL}}\right)$ and thoracic-right $\left(\mathrm{st}d_{\mathrm{TR}}\right)$, whereas the lower row contains lumbar-left $\left(\mathrm{st}d_{\mathrm{LL}}\right)$ and lumbar-right $\left(\mathrm{st}d_{\mathrm{LR}}\right)$ standard deviation values.

The bottom text in the class estimation is simply the natural language combination of all interpretations described above. The background colour follows the colour map for combined scoliosis and kyphosis/lordosis classes. In concrete cases, two patients have: 1. *Severe right-sided, mainly thoracic scoliosis* and 2. *Very significant left-sided thoraco-lumbar scoliosis with extreme kyphosis*.

The colour of the frontal and sagittal projection lines follows the colour map in Figure 5 and represent the mathematical curvature strain (20). For the frontal view, the colour scheme goes from green (normal spine segment) to yellow (extreme lateral curvature strain), while in the sagittal view, green represents the normal shape of the spine segment and magenta represents extreme front-back curvature strain. Extreme lateral curvatures and front-back curvatures are thus marked red. The thickness of the coloured segment is also an indication of the extreme curvature strain. The more extreme the curvature is, the larger the thickness of yellow or magenta becomes in the projections (or the shades of red in the combined strain).

The *ScolioClass* report can be customised and improved in various ways, e.g., all elements could be repositioned to make it as natural and intuitive as possible for medical professionals. The colour scheme can also be adjusted by simply adhering to a new colour map. Regarding the analysis, the boundaries can be easily redefined with new values, and the text interpretation could also be changed. This flexibility is valuable to allow medical professionals to customize the tool for their and their patients comfort and linguistic interpretations. But if the computed index values are preserved and reported, and if these values are compared across clinics, regardless of the linguistic interpretations, there is absolutely no risk of inconsistency.

For a more substantial improvement of the tool in the future, the estimation of the affliction could be refined by including other statistical moments and better using the angular orientations.

## 3 Results

### 3.1 Descriptive statistics

All 94 samples were processed using the *ScolioClass* MATLAB tool to reveal the severity of deformity and to calculate the indices described above. A basic frequency analysis of classes and subclasses is performed as well as a comparison against primary Cobb angles. The data reveal that most patients with AIS fall into the moderate to severe categories, with the highest prevalence in the “*very significant*” group (23 patients) and notable numbers in the “*slightly significant*” (20 patients) and “*extremely severe*” (20 patients) categories. In contrast, only a small number of patients are classified as having no scoliosis (7 patients) or mild scoliosis (10 patients). This distribution underscores the significant impact of scoliosis severity in the studied population, highlighting the need for careful monitoring and potential intervention for those affected.

Distribution of patients classified by the side of scoliosis using the S index: The data indicates that right-sided scoliosis is the most prevalent, affecting 31 patients, closely followed by left-sided scoliosis with 30 patients. Additionally, 25 patients have both-sided scoliosis, while only 8 patients are classified as having no scoliosis. This distribution suggests a significant predominance of right-sided and left-sided scoliosis in the population, emphasizing the need for targeted assessment and management strategies for these conditions.

Distribution of patients classified by the location of scoliosis using the *v* index: The data shows that the most common type of scoliosis in this population is “*mainly thoracic scoliosis*,” affecting 45 patients, followed by “*thoraco-lumbar scoliosis*” with 37 patients. In contrast, there are very few cases of lumbar scoliosis (1 patient), mainly lumbar scoliosis (3 patients), and thoracic scoliosis (1 patient). Additionally, 7 patients are classified as having no scoliosis in the frontal plane. This distribution highlights a significant prevalence of thoracic and thoraco-lumbar scoliosis.

Distribution of patients classified by the location of scoliosis using the kl index: The data indicates that “extreme kyphosis” is the most prevalent condition, affecting 25 patients, followed by “*slight kyphosis*” with 19 patients. Additionally, there are notable numbers of patients with “*slight lordosis*” (15 patients), “*significant lordosis*” (11 patients), and “*significant kyphosis*” (11 patients). Only 12 patients are classified as having “*extreme lordosis*” and just 1 patient has a “*normal spine*” in the sagittal plane.

### 3.2 Validation of *ScolioClass* classification method against Lenke classification scheme

**Figure 6:**
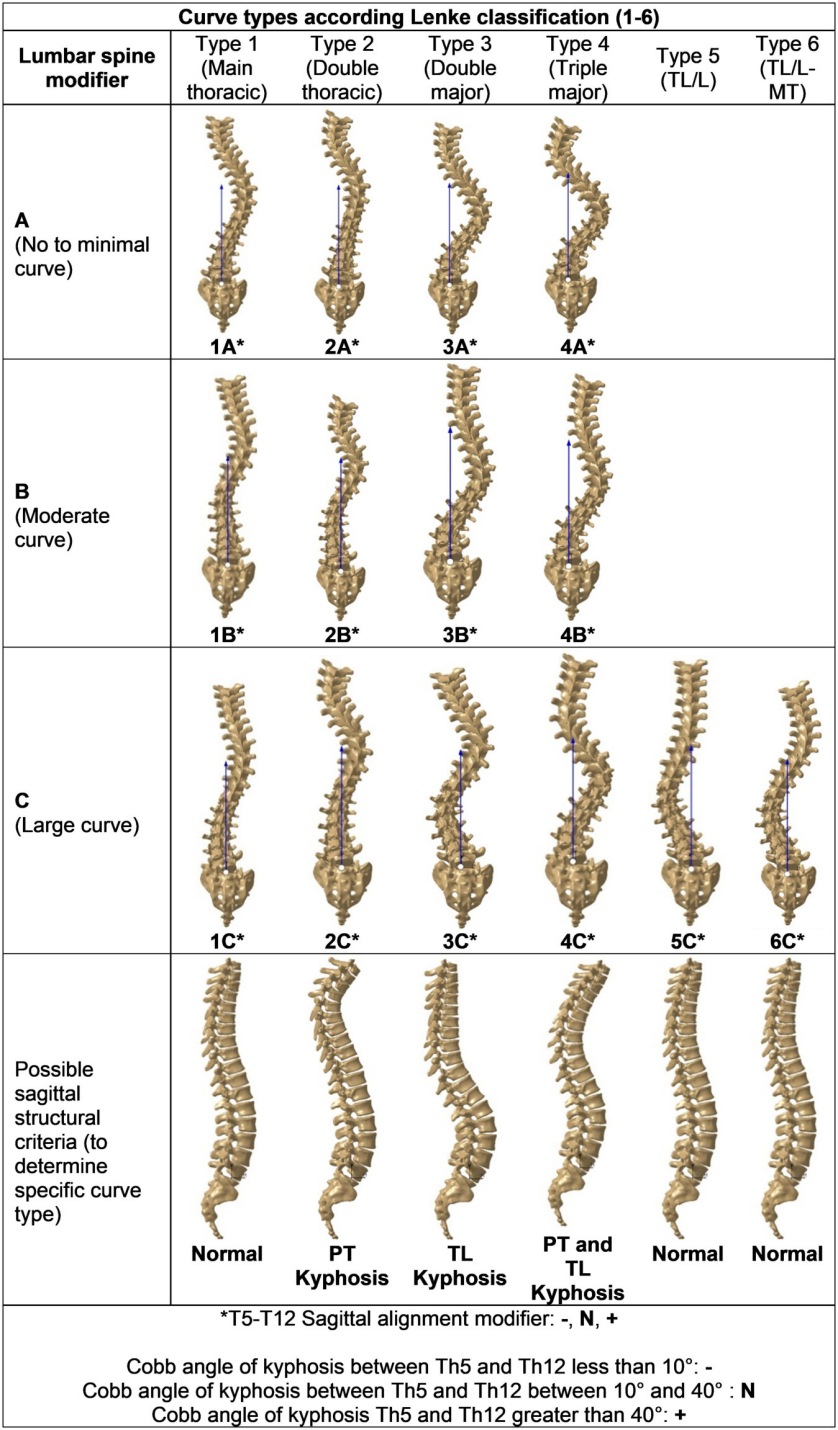
Lenke classification scheme.

To validate the *ScolioClass* classification method, we compared it with the Lenke classification (Figure 6) using the same dataset of 94 samples. In this context, the term “3D classification” refers to the use of continuous indices derived from three-dimensional spinal geometry (coronal, sagittal, and axial components) to describe scoliosis severity and type, in contrast to the Lenke system, which relies on categorical curve types and modifiers.

In the validation process, it is essential to recognize that the Lenke classification system is categorical system, emphasizes distinguishing between structural curves (rigid curves that remain above 25 degrees on bending x-rays and often necessitate surgical correction) and non-structural curves (more flexible, compensatory curves that typically do not require surgery) in specific anatomical regions: the Proximal Thoracic (T1-T3/T4), Main Thoracic (T4/T5-T12), and Thoracolumbar/Lumbar (T12-L4) areas.

*ScolioClass* by contrast, is a quantitative 3D system that utilizes AIS indices (Table 1) to quantify scoliosis severity through a numerical range of a continuous index (std), categorizing cases into: *No scoliosis*, *Mild scoliosis*, *Slightly significant scoliosis*, *Very significant scoliosis*, *Severe scoliosis*, and *Extremely severe scoliosis*. This approach does not rely on traditional Cobb angle measurements. In contrast, the Lenke classification does not define explicit severity ranges but instead differentiates structural and non-structural curves based on flexibility.

However, in the Lenke classification, lumbar spine modifiers A, B, and C are determined by the position of the lumbar curve's apical vertebra relative to the central sacral vertical line (CSVL): Modifier A indicates that the CSVL passes completely between the pedicles of the apical lumbar vertebra (suggesting a mild, well-balanced lumbar curve), modifier B is used when the CSVL touches the apical vertebra (indicating a moderate lumbar curve), and modifier C applies when the CSVL lies completely lateral to the apical lumbar vertebra (reflecting a more pronounced lumbar curve). In contrast, the *ScolioClass* classification does not include an adequate parameter to describe the apical vertebra's position relative to the CSVL, which meant that we were unable to perform a validation for this specific aspect of lumbar curve assessment.

Further comparison of the *ScolioClass* and Lenke classifications revealed that in the *ScolioClass* classification, the S Index is used to categorize scoliosis direction in left-sided scoliosis, both-sided scoliosis, and right-sided scoliosis, while Lenke classification does not use such direction-based categorization of scoliosis. On the other hand, *ScolioClass* emphasizes a more quantitative classification of the curve's vertical dominance based on the *v* -index that evaluates how much the curve involves either the thoracic or lumbar regions and can classify a curve as *Thoracic scoliosis*, *Mainly thoracic scoliosis*, *Thoraco-lumbar scoliosis or Lumbar scoliosis*, without segmenting specific vertebrae. Considering this, Table 2 presents the best possible correspondence between spinal regions as defined by the Lenke classification and the *ScolioClass* descriptions based on *v* -index values.

**Table 2:** Correspondence between spine regions in Lenke and *ScolioClass* description according to *v*-index.

Regions of the spine according Lenke	*ScolioClass* class description according to v-index
Proximal thoracic	Thoracic scoliosis
Main thoracic	Mainly thoracic scoliosis
Thoracolumar/Lumbar	Thoraco-lumbar scoliosis, Lumbar scoliosis or Mainly lumbar scoliosis

Based on Table 2, the following points should be addressed to validate the *ScolioClass* description according to *v* -index against the Lenke classification types:

1. Lenke type 1 (Main Thoracic scoliosis with structural curvatures in the Main Thoracic region) should correspond to Thoracic scoliosis or Mainly thoracic scoliosis in *ScolioClass*;
2. Lenke type 2 [Double Thoracic with structural curvatures in Proximal Thoracic region and Main Thoracic region (major)] should correspond to Thoracic scoliosis or Mainly thoracic scoliosis in *ScolioClass*;
3. Lenke type 3 [Double Major with structural curvatures in Main Thoracic (major) and Thoracolumbar/Lumbar region] should correspond to Mainly thoracic scoliosis or Thoraco-lumbar scoliosis in *ScolioClass*;
4. Lenke type 4 [Triple Major with structural curvatures in Proximal Thoracic, Main Thoracic (major) and Thoracolumbar/Lumbar region] should correspond to Mainly thoracic scoliosis or Thoraco-lumbar scoliosis in *ScolioClass*;
5. Lenke type 5 [Thoracolumbar/Lumbar with structural curvatures in Thoracolumbar/Lumbar (major) region] should correspond to Lumbar scoliosis, Mainly lumbar scoliosis or Thoraco-lumbar scoliosis in *ScolioClass*;
6. Lenke type 6 [Thoracolumbar/Lumbar – Main Thoracic with structural curvatures in Main Thoracic and Thoracolumbar/Lumbar (major) region] should correspond to Thoraco-lumbar scoliosis or Mainly lumbar scoliosis in *ScolioClass*.

To validate the *ScolioClass* description based on the v-index against the Lenke type, Table 3 includes four columns: Patient ID, which uniquely identifies each patient; *ScolioClass* Description, with the part based on the v-index shown in bold; Lenke Classification, with the Lenke type also in bold; and Validation Status, showing whether the *ScolioClass* description matches the Lenke type, based on the method described above.

**Table 3:** Part of the table for validating the *ScolioClass* classification against Lenke classification scheme.

Patient ID	*ScolioClass* description	Lenke classification	*ScolioClass* description based on *v*-index validation status	*ScolioClass* description according to the kl-index
1	Slightly significant left-sided thoracolumbar scoliosis with slight kyphosis.	6BN	Validated	Validated
2	Slightly significant left-sided thoracolumbar scoliosis with significant lordosis.	1BN	Not validated	Not validated
3	Normal spine.	Normal spine	Validated	Validated
…	…	…	…	
94	Very significant left-sided thoraco-lumbar scoliosis with extreme kyphosis.	5B+	Validated	Validated

Table 4 shows the total count of each Lenke type, the corresponding *ScolioClass* description based on the v-index, and the number of cases that were validated vs. not validated.

**Table 4:** Validated number in *ScolioClass* description based on *v*-index and total Lenke type number.

Lenke type	Lenke's type numbers	*ScolioClass* description based on *v*-index	Validated number	Not validated
Lenke type 1	8	Thoracic scoliosis or Mainly thoracic scoliosis	4	4
Lenke type 2	2	Thoracic scoliosis or Mainly thoracic scoliosis	2	0
Lenke type 3	33	Mainly thoracic scoliosis or Thoraco-lumbar scoliosis	29	4
Lenke type 4	2	Mainly thoracic scoliosis or Thoraco-lumbar scoliosis	2	0
Lenke type 5	9	Lumbar scoliosis, or Mainly lumbar scoliosis or Thoraco-lumbar scoliosis	9	0
Lenke type 6	15	Thoraco-lumbar scoliosis or Mainly lumbar scoliosis	13	2
Normal	25	Normal	9	16

We used two main metrics to assess the relationship between Lenke type and *ScolioClass* description based on *v* -index validation status. First, the Chi-square test (26) was employed to determine if the distribution of validated vs. not validated cases across different Lenke types deviated significantly from what would be expected under independence. Additionally, we calculated the overall percentage agreement (27), which provides a simple, broad overview of validation rates, though it does not capture the nuanced differences between the various Lenke types.

The Chi-square test yielded a statistic of approximately 29.0 with 6 degrees of freedom and a *p*-value of less than 0.0001, demonstrating a statistically significant association between Lenke type and *ScolioClass* description based on *v* -index. Additionally, the overall percentage agreement indicated that about 72.3% of cases were validated, providing a broad overview of the data distribution across all categories.

**Figure 7:**
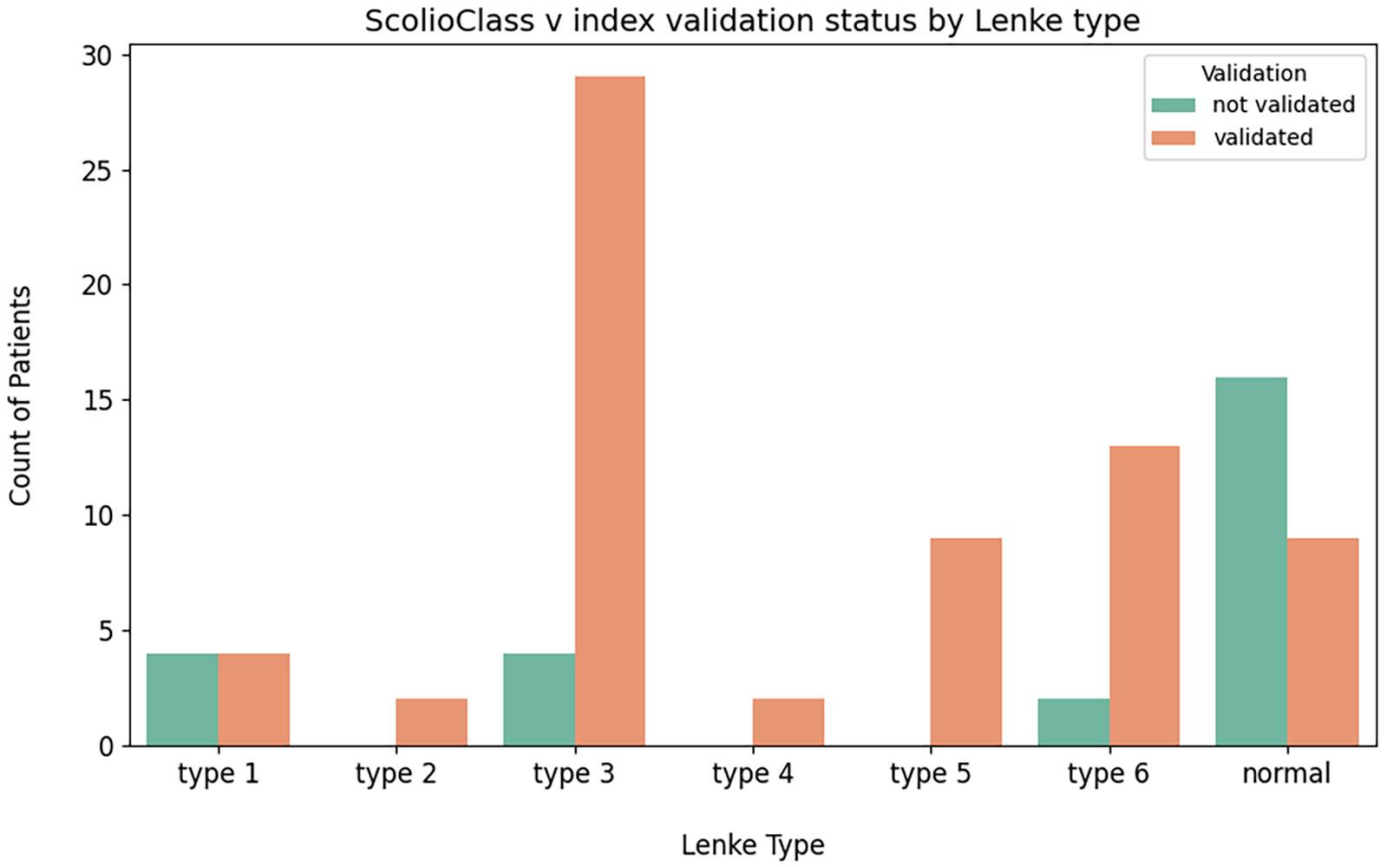
Stacked bar chart of validated vs. not validated *ScolioClass* descriptions based on *v*-index for each Lenke type.

**Figure 8:**
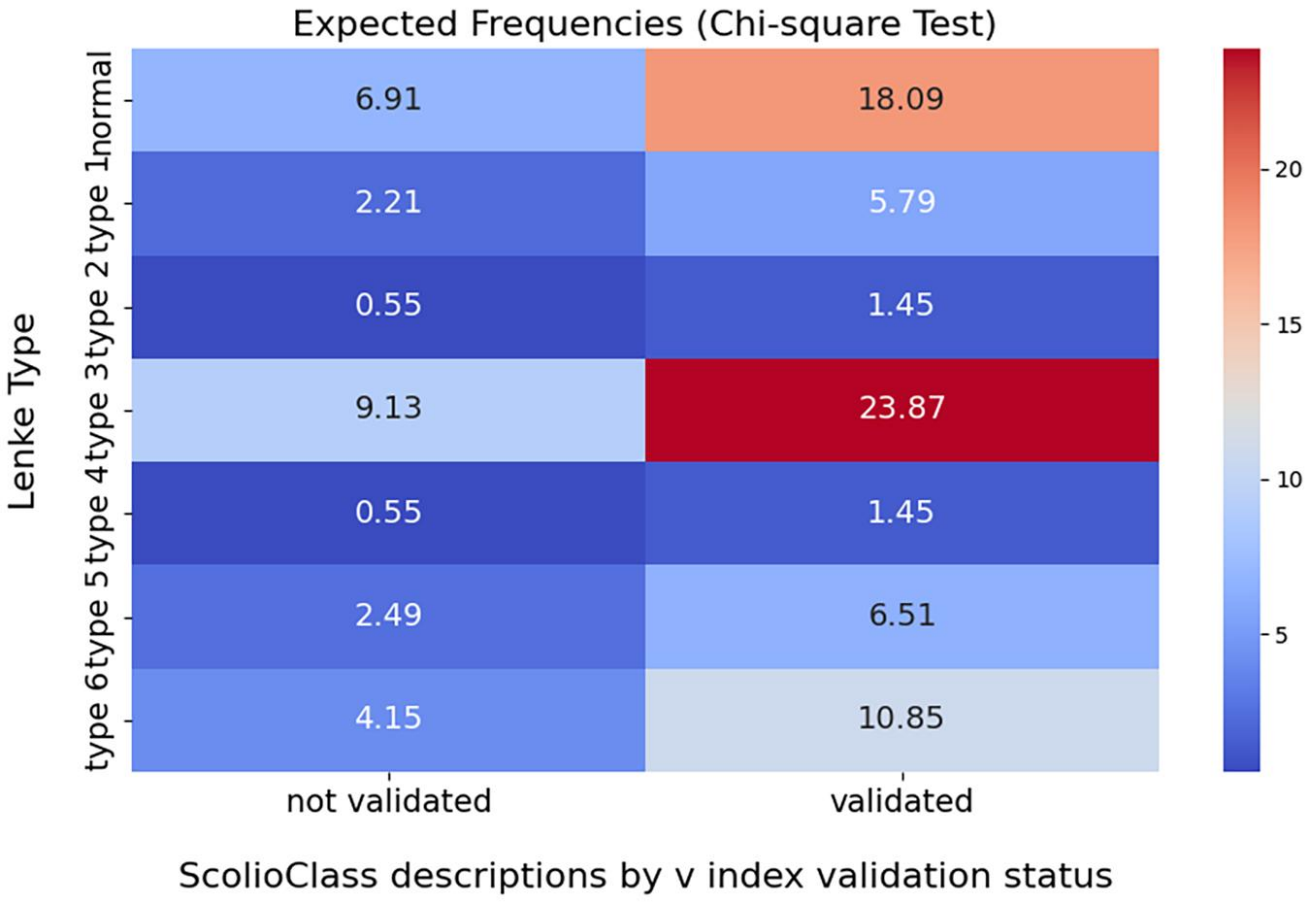
Heat map of expected frequencies of validated and not validated *ScolioClass* descriptions by v-Index for each Lenke type.

Additionally, grouped bar charts in Figure 7 display the number of validated vs. not validated cases for each Lenke type, allowing a visual comparison of the distribution of the *ScolioClass* description based on the v-index across these groups. In contrast, the heat map in Figure 8 visualizes the expected frequencies from the Chi-square test, highlighting areas where the actual data deviates from what would be expected under the null hypothesis. Together, these visualizations underscore the statistically significant association, and the overall validation rate illustrating how different Lenke types align with *ScolioClass* outcomes.

The contingency Table 4 shows the distribution of validation status across different Lenke types, revealing those certain types, specifically types 3 and 6, have higher counts of validated cases, while cases labelled as “normal” tend to have lower validation frequencies. The grouped bar chart further emphasizes these differences, providing a clear visual of how validation status for the *ScolioClass* description based on *v* -index varies by Lenke type. Additionally, the heatmap comparing observed and expected frequencies from the Chi-square test demonstrates notable deviations, particularly for the “normal” category and some specific Lenke types.

The *ScolioClass* kl index offers a detailed, quantitative measure of sagittal balance, with positive values indicating kyphosis and negative values representing lordosis, classified into seven categories: Extreme lordosis, Significant lordosis, Slight lordosis, Normal spine, Slight kyphosis, Significant kyphosis, and Extreme kyphosis. In contrast, the Lenke classification with ±*N* signs provides a binary evaluation of kyphosis, with + indicating excessive kyphosis, - indicating *ScolioClass*, N indicating normal kyphosis, without providing information about the extent or severity of the kyphosis or any assessment of lordosis.

Based on the above-mentioned, we can conclude the following:

1. +sign in Lenke classification should correspond to Significant kyphosis, and Extreme kyphosis in *ScolioClass*.
– sing in Lenke has no correspondence in *ScolioClass*.
3. The N sign in Lenke should correspond to Normal spine, spine with slight kyphosis, and spine with slight lordosis in *ScolioClass*.
4. /- No Lenke correspondence to Extreme lordosis, Significant lordosis.

Table 3 is also used for validating the *ScolioClass* description according to the kl-index against Lenke's sagittal alignment modifier. It contains four columns: Patient ID, which uniquely identifies each patient; full *ScolioClass* Description, where the *ScolioClass* description according to the kl-index is underlined; Lenke classification, where Lenke's sagittal alignment modifier is underlined; and *ScolioClass* description according to the kl-index validation status. The validation is done according to the method described above.

 Table 5 shows the validated number in the *ScolioClass* description according to the kl-index and Lenke's sagittal alignment modifier number. The Lenke classification does not specifically identify cases of lordosis. In contrast, *ScolioClass* identifies 16 cases of extreme or significant lordosis, highlighting its ability to provide a more nuanced classification of spinal curvature types, particularly regarding lordosis. However, there are two cases in the Lenke classification that do not correspond to any *ScolioClass* data. These cases represent atypical instances of reduced kyphosis that *ScolioClass* did not categorize, suggesting potential gaps in cross-classification for this spine type.

**Table 5:** Validated number in *ScolioClass* description based on kl-index and Lenke's sagittal alignment modifiers (±*N* signs).

Lenke's sagittal alignment modifier	Lenke's sagittal alignment modifier number	*ScolioClass* description according to kl-index	Validated number	Not Validated number
+	31	Significant kyphosis and Extreme kyphosis	28	3
−	2	/	0	2
*N*	45	No kyphosis, Slight kyphosis, Slight lordosis	36	9
/	16	Extreme lordosis, Significant lordosis	0	16

A Chi-square test was conducted to evaluate the association between Lenke's sagittal alignment modifiers and *ScolioClass* description based on the kl-index. The analysis revealed a statistically significant relationship (*χ*^2^ = 48.39, df = 3, *p* < 0.0001), indicating that validation outcomes are not independent of the Lenke modifier. Out of 94 cases, 64 were validated, resulting in an overall percentage agreement of 68.09%. Specifically, the “+” and “*N*” modifiers showed high validation rates, whereas the “–” modifier and the “/” symbol, indicating no Lenke modifier for lordosis cases, were predominantly not validated.

**Figure 9:**
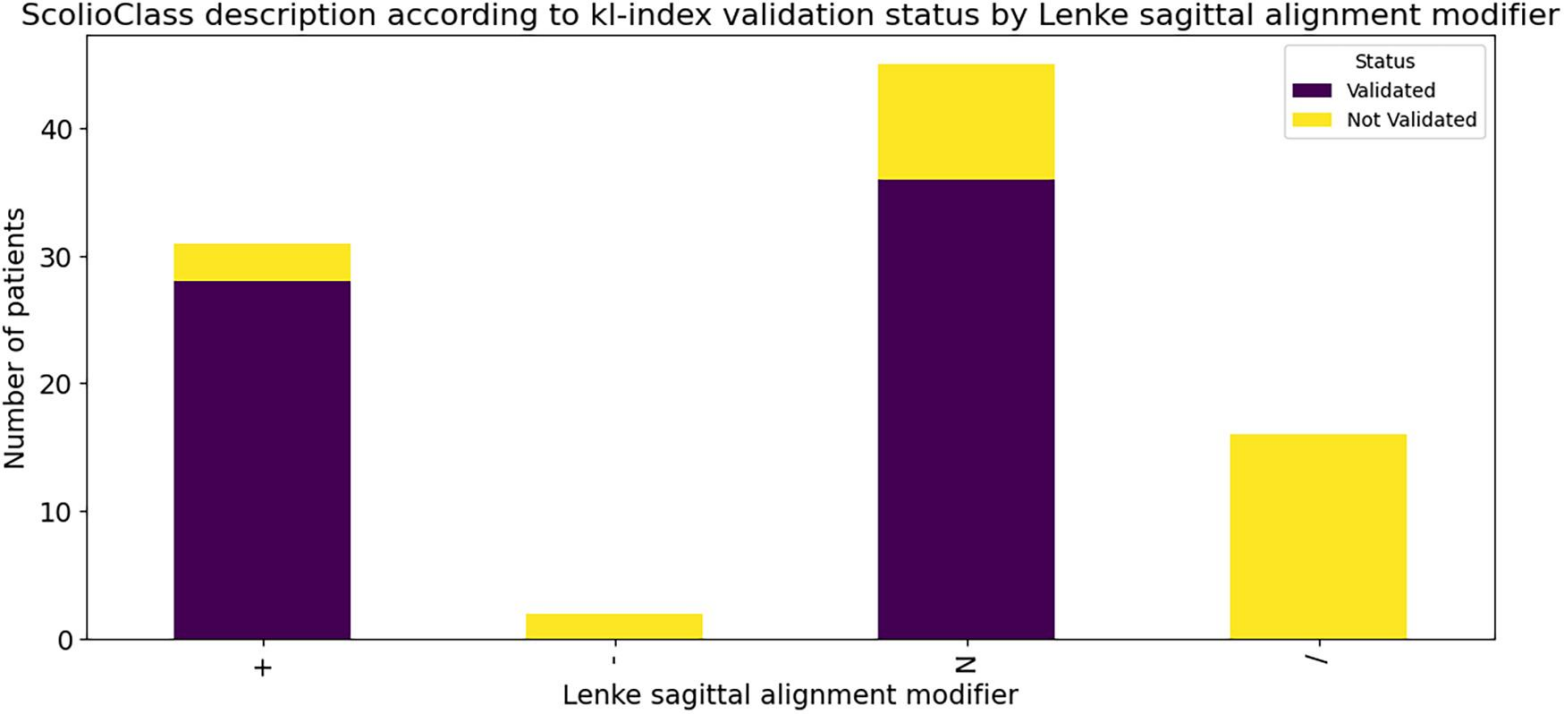
Stacked bar chart of validated vs. not validated and *ScolioClass* description based on kl-index for each Lenke sagittal alignment modifier.

**Figure 10:**
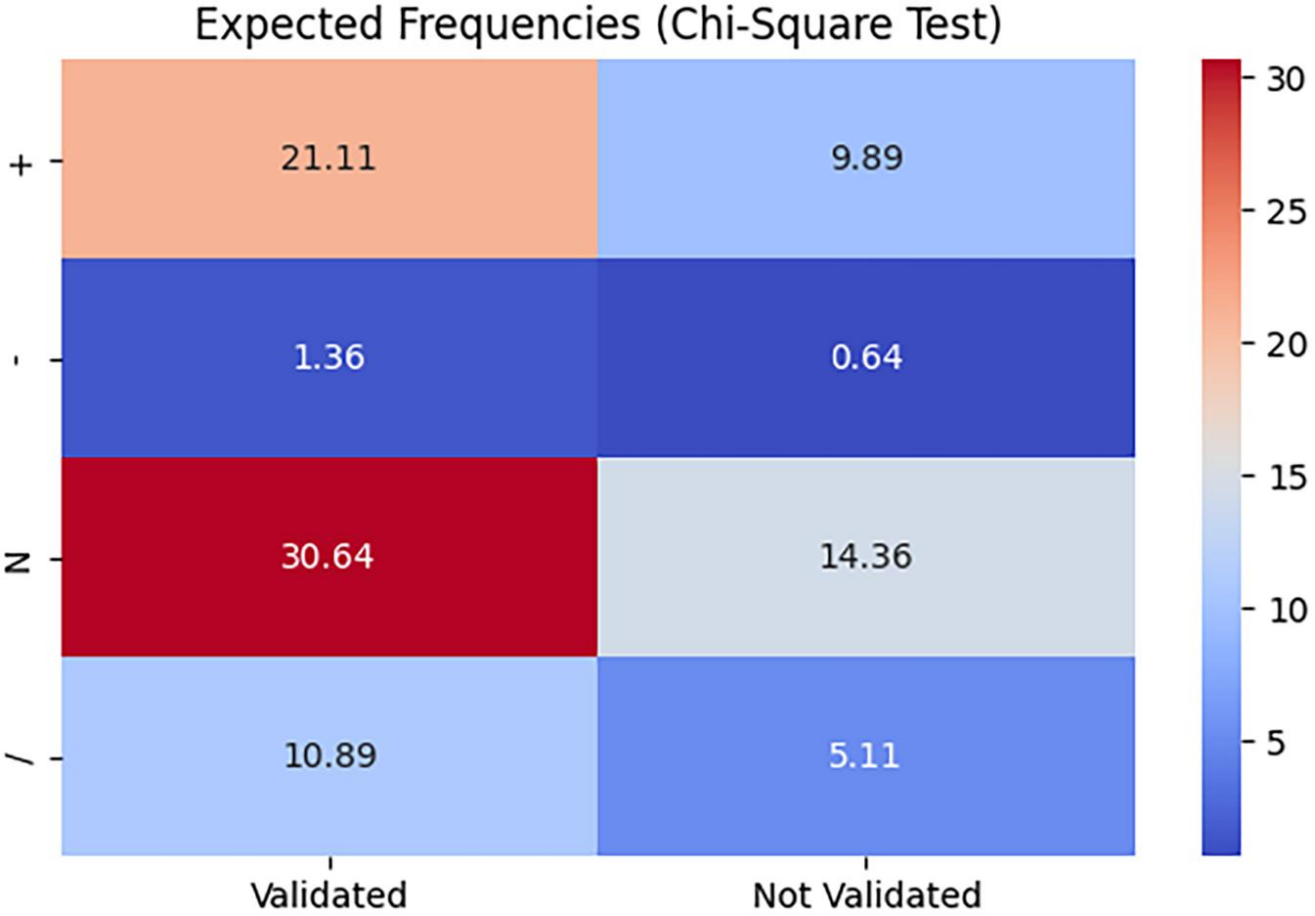
Heat map of expected frequencies of validated vs. not validated *ScolioClass* descriptions based on the kl-index for each Lenke sagittal alignment modifier and the “/” symbol, indicating no Lenke modifier for lordosis cases.

A stacked bar chart (Figure 9) illustrated these differences clearly, and a heat map of expected frequencies (Figure 10) highlighted substantial deviations from expected values under the null hypothesis, supporting the significance of the Chi-square result.

## 4 Discussion

The present study investigated the relationship between Lenke classification types and the validation outcomes of corresponding *ScolioClass* descriptions in a cohort of 94 adolescent idiopathic scoliosis (AIS) patients. By employing a contingency table approach alongside Chi-Square testing and percentage agreement measures, we were able to assess how well a novel, continuous-index–based system (*ScolioClass*) aligns with the widely used, treatment-oriented Lenke classification. In this context, *ScolioClass* represents a “3D classification” framework because it integrates continuous indices that capture coronal, sagittal, and axial deformity components in three dimensions, whereas Lenke relies on categorical curve types and modifiers derived from 2D radiographs.

*ScolioClass* utilizes quantitative indices—namely the AIS index and S Index—derived from 3D optical scans to capture both curvature severity and directionality without relying on Cobb angle measurements. In contrast, the Lenke system categorizes curves based on Cobb angles and structural flexibility, using apex positioning and lumbar modifiers (A, B, C) relative to the central sacral vertical line (CSVL) to guide surgical decision-making. This highlights a fundamental difference: *ScolioClass* applies continuous, data-driven thresholds, while Lenke applies discrete anatomical rules. This fundamental difference underlies many of the observed discrepancies between the two systems.

Statistical analysis revealed a significant association between Lenke type and *ScolioClass* validation status (*χ*^2^ ≈ 29.0, df = 6, *p* < 0.001), and an overall agreement rate of 72.3%. While this demonstrates substantial concordance, analysis of the “normal” Lenke group—where Cobb angles < 10° are excluded—highlighted a higher non-validation rate. This suggests that *ScolioClass* detects subtle deformities overlooked by Lenke criteria, underscoring its sensitivity to mild curves. In practical terms, *ScolioClass* expands the classification spectrum to include gradations of severity that are not captured within Lenke's categorical framework.

In addition to curve types, concordance between sagittal alignment modifiers and the *ScolioClass* kl-index was 68.1%, also statistically significant (*χ*^2^ ≈ 48.39, df = 3, *p* < 0.0001). These levels of concordance highlight that while the two systems are related, differences are largely explained by their conceptual foundations: Lenke relies on categorical curve rules with a binary sagittal modifier, whereas *ScolioClass* applies continuous indices that capture a wider spectrum of deformities, including lordosis and subtle curvatures not defined in Lenke. Thus, the observed concordance validates the overall alignment between the systems while demonstrating *ScolioClass's* capacity to provide finer granularity.

Further evaluation of sagittal alignment modifiers showed that cases with significant kyphosis (“+”) and normal curvature (“*N*”) achieved high validation rates (28/31 and 36/45, respectively), whereas lordotic (“–”) and unclassified (“/”) cases exhibited poor concordance (0/16 validated). A Chi-Square test across modifiers confirmed these differences were highly significant (*χ*^2^ ≈ 48.39, df = 3, *p* < 0.0001). The inability of the Lenke system to explicitly categorize lordosis may explain the zero-validation outcome in the “/” category, whereas *ScolioClass's* kl-index identified 16 lordosis cases, highlighting its capacity for nuanced sagittal profiling. This again reflects the contrast between Lenke's binary sagittal categorization and *ScolioClass's* graded, continuous approach.

Several methodological factors contribute to the observed divergences. *ScolioClass* thresholds are currently set by simple cutoffs for informational purposes rather than mirroring Lenke's clinical criteria. Moreover, *ScolioClass* does not yet incorporate a lumbar modifier analogous to Lenke's A–C scheme, limiting direct comparison in lumbar assessments. These differences partly account for atypical cases—such as reduced kyphosis—not covered by *ScolioClass* categories.

Despite these limitations, *ScolioClass* offers several advantages:

- Non-invasive assessment: By using 3D optical scanning, it avoids repeated x-ray exposure and enables more frequent monitoring.
- Continuous, quantitative grading: The AIS and S indices provide a finer granularity of severity and directional information, which may enhance automated analysis in research and clinical imaging.
- Detection of subtle and combined deformities: Its ability to identify mild curvatures and combined kyphosis–scoliosis patterns offers broader coverage of spinal deformity types.

Nonetheless, the clinical significance of these additional classification dimensions requires further validation. Future work should refine *ScolioClass* thresholds through statistical calibration (e.g., distribution-based measures and ROC curve analysis) and integrate a lumbar modifier to better correspond to Lenke's structural criteria. Prospective clinical studies will be essential to determine how enhanced granularity impacts treatment planning, patient outcomes, and the risk-benefit balance of reduced radiation exposure. In addition, assessing correlations with clinical endpoints, including surgical planning decisions, longitudinal curve progression, and patient-reported outcome measures, will be critical to demonstrate the broader clinical utility of the system. Although the present study is limited by the relatively small dataset of 94 cases from a single institution, ongoing efforts to expand the cohort across multiple centres and more diverse populations are expected to enhance external validity, support the statistical optimization of threshold settings, and establish the generalizability and international applicability of the proposed framework. It is also noted that the gender imbalance of the present cohort reflects the higher prevalence of AIS in females (2). Nevertheless, both sex distribution and age effects will warrant further evaluation in larger, demographically balanced studies.

In recent years, a variety of non-invasive methods have been developed to complement or replace radiographic assessment of scoliosis. Below, *ScolioClass* is compared to selected approaches, with an emphasis on data input, classification scope, accuracy, methodological strengths and weaknesses, and overall clinical suitability. This situates *ScolioClass* as a potential bridge between basic screening tools and high-precision diagnostics in the current clinical environment, where radiation reduction and artificial intelligence (AI) integration are priorities.

One prominent category of techniques is 3D markerless surface topography (ST) combined with multi-task learning (MTL) (28, 29). By applying fringe projection profilometry for 3D torso reconstruction and convolutional neural networks for angle prediction and type classification, these systems have achieved high sensitivity (96%–99%) for curves exceeding 25°, with correlations of *r* = 0.7–0.9 to Cobb angles in samples of more than 165 patients. While comparable to *ScolioClass* in terms of radiation-free data acquisition, this approach primarily targets external asymmetry and is limited in linking internal and external deformity features. *ScolioClass*, in contrast, incorporates sagittal balance indices (e.g., kl index) and therefore provides a more comprehensive anatomical characterization, although ST may be more practical for large-scale population screening.

Least-squares support vector machines (LS-SVM) applied to 3D back surface data represent another non-invasive strategy. Modified LS-SVM classifiers using custom kernels have achieved 95% accuracy and kappa values of 0.86–0.91 in classifying curve types, including thoracic, double major, and lumbar/thoracolumbar patterns. Although highly effective for morphological categorization, these systems provide less detail regarding curve severity. *ScolioClass* demonstrates added value by quantifying severity across six discrete levels and by assessing sagittal balance with validated agreement to Lenke classifications. Furthermore, LS-SVM systems typically require extensive training datasets, whereas the spline interpolation framework used in *ScolioClass* is comparatively more accessible for routine clinical deployment.

Traditional methods such as the scoliometer and Moiré topography remain widely used for initial screening. These approaches achieve moderate correlations with radiographic Cobb angle (*r* = 0.6–0.8) and sensitivities of approximately 83% for curves above 20° but are subject to significant inter-operator variability (30, 31). Although inexpensive and rapid, they lack the quantitative rigor and reproducibility required for advanced diagnostics. *ScolioClass* addresses these limitations by offering automated, reproducible three-dimensional indices, thereby providing higher diagnostic validity beyond simple triage.

Other methods focus more directly on therapeutic applications, such as Schroth and Rigo curve-type classifications enhanced by machine learning (32). These physiotherapy-oriented systems achieve reliability scores around 88% (kappa = 0.86) and are particularly effective in guiding individualized exercise regimens (33). While *ScolioClass* is less therapy-focused, its objective metrics, including natural-language reporting, make it more suitable for diagnostic workflows while still providing complementary information for treatment planning.

Taken together, these comparisons position *ScolioClass* as a method that balances accessibility and diagnostic precision. While many radiation-free approaches emphasize external morphology, *ScolioClass* integrates both external and internal indices, offering superior sagittal characterization and alignment with established clinical classifications. Its compatibility with existing workflows and quantitative rigor suggest that it can serve as a bridge between rapid, large-scale screening methods and highly specialized diagnostic tools, supporting ongoing clinical efforts to minimize radiation exposure and advance AI-driven scoliosis management.

## 5 Conclusions

In summary, this comparative analysis demonstrates that *ScolioClass* correlates strongly with Lenke classification overall, while offering greater sensitivity to subtle and sagittal deformities. Although current evidence is limited by sample size, uneven subgroup distribution, and potential inter-operator variability, methodological refinements and expanded multi-center validation are expected to improve reproducibility and generalizability. With methodological refinements and clinical validation, *ScolioClass* has the potential to complement or even supplant traditional AIS classification paradigms.

## Data Availability

The data analyzed in this study is subject to the following licenses/restrictions: the participants of this study did not give written consent for their data to be shared publicly, so due to the sensitive nature of the research, supporting data is not available. Requests to access these datasets should be directed to Tito Bassani, tito.bassani@grupposandonato.it.

## Appendix

**Table A1:** This table contains a glossary and short description of the index names used in the section 2.3.3.

$\mathrm{STD}=\left[\begin{array}{cc} \mathrm{st}d_{\mathrm{TL}} & \mathrm{st}d_{\mathrm{TR}}\\ \mathrm{st}d_{\mathrm{LL}} & \mathrm{st}d_{\mathrm{LR}} \end{array}\right]$	Standard deviation (std) of the frontal projection interpolated curve, computed for points in the thoracic-left (std_TL_), thoracic-right (std_TR_), lumbar-left (std_LL_) and lumbar-right (std_LR_)
*s*	Sidedness index, indicating how much the frontal projection std values lean towards the left or right of the expected vertical axis.
*v*	Vertical index, indicating how much the frontal projection std values lean towards the thoracic or lumbar section of the spine.
$\mathrm{kl}=\frac{m_{T}+m_{L}}{m_{T}-m_{L}}$	The kyphosis/lordosis index, computed from the statistical means for the thoracic $m_{T}$ and lumbar $m_{L}$ regions, in the sagittal projection
